# Induction of pyroptotic cell death as a potential tool for cancer treatment

**DOI:** 10.1186/s12950-022-00316-9

**Published:** 2022-11-14

**Authors:** Sara Socorro Faria, Anuruddika Jayawanthi Fernando, Vladmir Cláudio Cordeiro de Lima, Adriano Giorgio Rossi, Juliana Maria Andrade de Carvalho, Kelly Grace Magalhães

**Affiliations:** 1 Laboratory of Immunology and Inflammation, Department of Cell Biology, University of Brasilia, DF Brasilia, Brazil; 2 Edinburgh BioQuarter, University of Edinburgh Centre for Inflammation Research, Queen’s Medical Research. Institute, University of Edinburgh, Edinburgh, UK; 3 Department of Medical Oncology, A.C. Camargo Cancer Center, Sao Paulo, SP Brazil

**Keywords:** Pyroptosis, cancer, Cell death, Chemotherapy, Immunotherapy, Inflammation

## Abstract

Cancer is a complex pathological disease and the existing strategies for introducing chemotherapeutic agents have restricted potential due to a lack of cancer cell targeting specificity, cytotoxicity, bioavailability, and induction of multi-drug resistance. As a prospective strategy in tackling cancer, regulating the inflammatory pyroptosis cell death pathway has been shown to successfully inhibit the proliferation and metastasis of various cancer cell types. Activation of inflammasomes such as the NLRP3 results in pyroptosis through cleavage of gasdermins, which forms pores in the cell membranes, inducing membrane breakage, cell rupture, and death. Furthermore, pyroptotic cells release pro-inflammatory cytokines such as IL-1β and IL-18 along with various DAMPs that prime an auxiliary anti-tumor immune response. Thus, regulation of pyroptosis in cancer cells is a way to enhance their immunogenicity. However, immune escape involving myeloid-derived suppressor cells has limited the efficacy of most pyroptosis-based immunotherapy strategies. In this review, we comprehensively summarize the cellular and molecular mechanisms involved in the inflammasome-mediated pyroptosis pathways in cancer cells, exploring how it could modulate the tumor microenvironment and be beneficial in anti-cancer treatments. We discuss various existing therapeutic strategies against cancer, including immunotherapy, oncolytic virus therapy, and nanoparticle-based therapies that could be guided to trigger and regulate pyroptosis cell death in cancer cells, and reduce tumor growth and spread. These pyroptosis-based cancer therapies may open up fresh avenues for targeted cancer therapy approaches in the future and their translation into the clinic.

## Background

Our innate immune system detects and engages with pathogen-associated molecular patterns (PAMPs) using pattern-recognition receptors (PRRs) expressed either on the surface or intracellularly, to remove the invading pathogens [1]. PRRs including Toll-like receptors (TLRs) and C-type lectin receptors (CLRs) located on the surface of immune cells directly sense PAMPs and damage-associated molecular patterns (DAMPs) [2]. However, NLRs (Nucleotide-binding oligomerization domain (NOD) and leucine-rich repeat (LRR) receptors), RLRs (retinoic acid-inducible gene I (RIG-I)-like receptors), and ALRs (absent in melanoma 2 (AIM2)-like receptors) are PRRs that are located intracellularly [3].

In particular, NLRs and ALRs have distinct domain architectures and functions [4]. Upon activation, NLRs assemble to form large molecular protein complexes within the cytoplasm referred to as inflammasomes [5]. The inflammasome is composed of NLR or ALR and a bipartite protein called ASC (apoptosis-associated speck-like protein containing a caspase-activation and recruitment domain) which binds to caspase-1 and directly activates it [6]. Upon activation, caspase-1 cleaves the precursor forms of IL-1β and IL-18 into their active forms. Furthermore, it cleaves Gasdermin D (GSDMD) to its active N-terminal fragment which forms pores in the plasma membrane resulting in a type of inflammatory cell death called pyroptosis [7–9].

Dysregulated inflammatory response may lead to the initiation and progression of tumors by providing growth factors and DNA-damaging agents for enhanced DNA replication and genomic instability [10]. The pyroptosis-dependent canonical NLRP3 inflammasome pathway plays a dichotomous part in tumor initiation. In various cancer experimental models, the NLRP3-pyroptosis axis induces tumor initiation and progression, supporting the expansion of myeloid-derived suppressor cells (MDSCs) in the tumor microenvironment (TME) [11, 12] On the other hand, NLRP3 inflammasome activation and pro-inflammatory cytokine release by dendritic cells responding to DAMPs released from dying tumor cells are important in priming CD8^+^ T cells, thereby enhancing antitumor immunity [13]. These activities create an inflamed and immunosuppressive microenvironment in which the tumor cells can thrive [14–16].

In this review, we provide an in-depth overview of the different roles of pyroptosis in cancer. Both immune and non-immune functions of inflammasome-dependent pyroptosis may happen in parallel or independently, depending on the cancer type. Therefore, it is essential to understand the molecular mechanisms by which this pathway contributes to cancer. Here we further discuss an up-to-date perspective on the clinical relevance of pyroptosis and its potential as a therapeutic target in cancer therapy.

### Pyroptosis pathways: canonical and non-canonical inflammasome signaling

Pyroptotic cell death takes the form of regulated necrosis, however, in some instances, the pathway can also engage apoptosis. Pyroptosis is the result of GSDMD being cleaved into its N-terminal fragment, which can then oligomerize to form pores in the cell membrane aiding the release of pro-inflammatory cytokines [17, 18]. To date, the human GSDM family is comprised of six genes (gasdermin A [*GSDMA*], gasdermin B [*GSDMB*], gasdermin C [*GSDMC*], gasdermin D [*GSDMD*], gasdermin E [*GSDME*], also known as *DFNA5*) and *pejvakin* (*PJVK*) [19]. Among these, GSDMD is the main gasdermin responsible for inflammasome-induced pyroptosis [20, 21]. GSDMD is expressed principally in macrophages and DCs [21] and is transcriptionally controlled by interferon regulatory transcription factor-2 (IRF2) [22]. In cancer, loss of IRF2 and IRF1 expression leads to immune evasion, representing a regulatory axis in cell death [23].

Gasdermins can also be cleaved by apoptotic caspases such as caspase-3, caspase-6, caspase-8, granzymes, death-related proteases in natural killer cells (NK cells), and cytotoxic T lymphocytes (CTLs) [24–26]. In this sense, GZMB cleaves GSDME in the same region as caspase-3, while GSDMB can be cleaved by GZMA [27, 28]. In addition, active caspase-8 can cleave GSDMD to initiate pyroptosis [29], a process that is inhibited by cFLIP_L_ [30]. Furthermore, in cancer cells, caspase-8 which is activated by macrophage-derived TNF-α can then cleave GSDMC, thus switching apoptosis into pyroptotic cell death [31].

During canonical pyroptotic pathway activation, the GSDMD-NT fragment moves toward the plasma membrane to form pores, resulting in cell depolarization and cytokine secretion [32]. GSDMD cleavage also forms GSDMD-NT pores on mitochondrial membranes releasing mitochondrial DNA (mtDNA) which could activate cyclic guanosine monophosphate-adenosine monophosphate synthase (cGAS), thereby activating the inflammasomes [33]. Activated cGAS can also promote the conversion of ATP and GTP into cyclin GMP-AMP (cGAMP), which in turn binds to and activates STING [34].

**Fig. 1:**
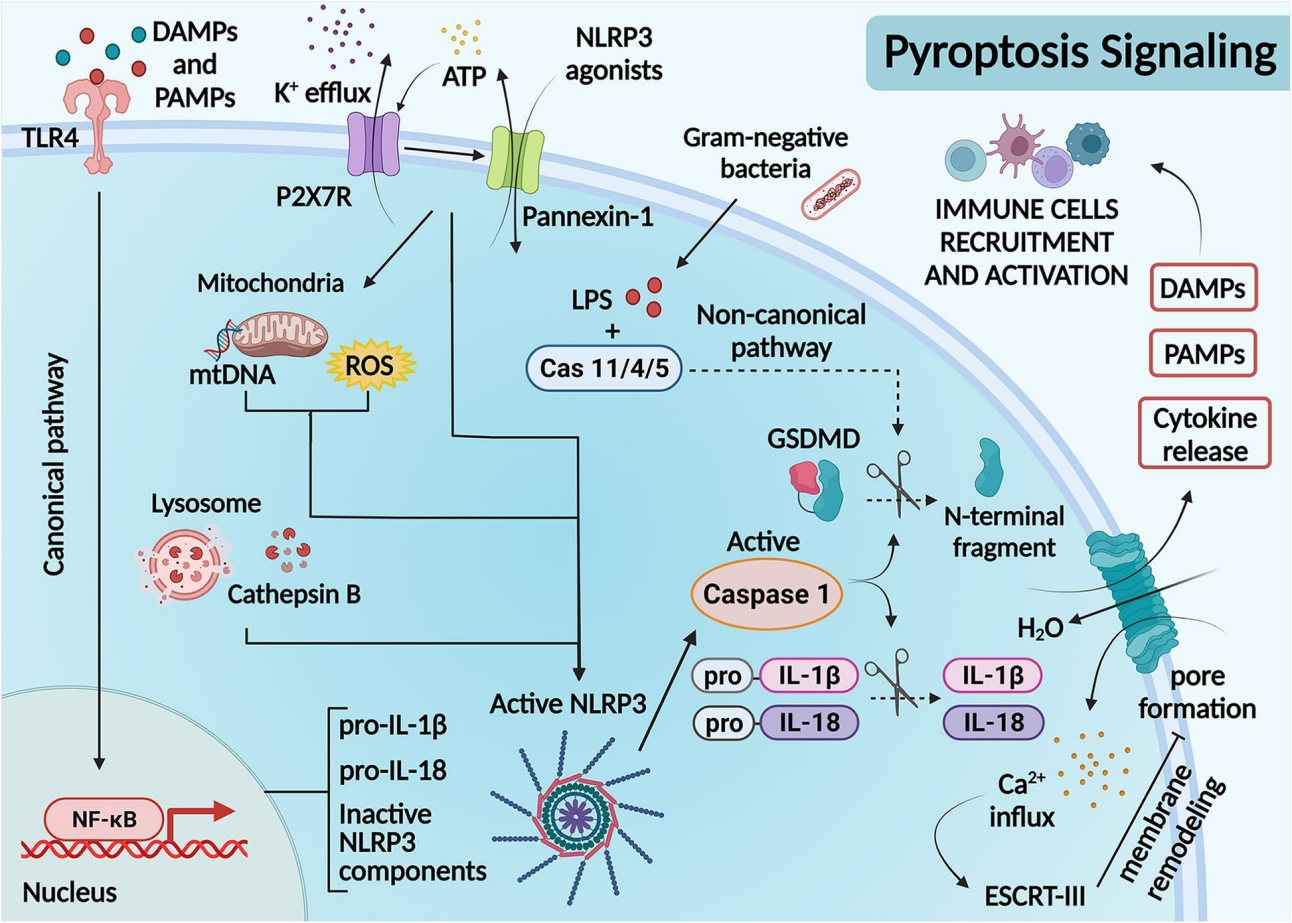
Mechanisms of canonical and non-canonical pyroptotic cell death. Canonical pyroptotic cell death activation is triggered in response to various stimuli. The first signal (*priming step*) is initiated through the identification of PAMPs and DAMPs by the pattern recognition receptors (PRRs) including toll-like receptors (TLRs). This first signal activates the NF-κB transcription factor, which encodes for pro-IL-1β, IL-18, and inactive NLRP3 components. Subsequently, other agonists act as the second signal (activation step), for example; extracellular ATP sensing that activates P2 × 7 receptors, leading to potassium efflux and activating Pannexin-1, allowing intracellular ATP release. These alterations can trigger an accumulation of mitochondrial reactive oxygen species (ROS), mitochondrial membrane permeabilization, and cathepsin B release by damaged lysosomal compartments. These signals stimulate NLRP3 inflammasome assembly and activation, inducing caspase-1 self-cleavage, resulting in IL-1β and IL-18 maturation and subsequent cleavage of Gasdermin D (GSMD). The N-terminal GSDMD domain forms membrane pores, releasing intracellular components, such as cytokines, PAMPs, and DAMPs, resulting in the recruitment of immune cells and their activation. Conversely, non-canonical activation requires direct binding of caspase-4/5 (in humans) or caspase-11 (in mice) to cytosolic lipopolysaccharides. Active caspase then cleaves GSDMD leading to pyroptosis. A variety of molecules can be modulated to induce or inhibit any step of this signaling cascade. As an example, ESCRT-III is modulated upon certain conditions as an attempt to remodel the membrane pores and diminish both pyroptosis and cytokine secretion, being an attractive target for regulating pyroptosis in different models

GSDMD pore formation in the plasma membrane also allows Ca^2+^ influx from the extracellular environment, which can direct calpain activation and ESCRT (endosomal sorting complex required for transport) assembly. Membrane repair mediated via ESCRT downstream of activated GSDMD can negatively regulate inflammasome-induced pyroptosis [35]. Failure to repair these pores will result in pyroptosis and pro-inflammatory cytokine and alarmins release, further enhancing the inflammatory response (Fig. 1).

During infection, cytosolic lipopolysaccharides (LPS) are detected by human caspases 4 and 5 and murine caspase-11, to induce non-canonical activation of the NLRP3 inflammasome pathway [36, 37]. Before activation, caspase-11 is primed by either type I interferon or IFN-γ [38, 39]. Following LPS stimulation, active caspase-11 can modify Pannexin-1, resulting in extracellular ATP influx through the P2 × 7-receptor ion channels, thereby inducing pyroptosis [40]. In some tumor models (melanoma and leukemia), extracellular ATP activates the P2 × 7-NLRP3-inflammasome pathway, driving macrophage pyroptosis and enhancing the maturation and antigen presentation capacity of DCs [41].

As mentioned earlier, the GSDMD amino-terminal fragment can locate the mitochondria to result in mitochondrial outer membrane permeabilization, thus promoting caspase-3 activation [42]. This is supported by complex mitochondrial crosstalk between gasdermins D and E [43] in an inflammasome-dependent way [44]. This shows that when both GSDMD and GSDME are present, they do not necessarily have to follow GSDMD cleavage to initiate cell death or cytokine release [45]. Interestingly in lung cancer, the mitochondria-mediated apoptotic pathway and its interconnected pyroptotic signal, are both important for tumor cell clearance as seen by a concordant cleavage pattern of both caspase-3 and GSDME [46].

Through this mechanism, the expression of GSDME increases phagocytosis of cancer cells and recruitment of CD8^+^ T cells, resulting in a positive feedback loop in a caspase-independent manner [47]. *Gsdme* gene silencing in melanoma cells has been shown to reduce pyroptotic cell death and resulting immune infiltrate [48], thus boosting anti-cancer immunity under experimental conditions [49]. Together, a comprehensive understanding of GSDME function in different types of cancer is worthy of further investigation.

### Inflammasome-pyroptosis axis: broader role in TME

Inflammasomes are multimeric protein complexes that are formed within the cytoplasm of cells and they regulate inflammatory responses and pyroptosis [50, 51]. The main PRRs for assembling the inflammasome complex include NLRP1 and 3, NLRC4, AIM2, and pyrin [52]. Other innate receptors like NLRP6, NLRP12, interferon-γ-inducible protein 16 (IFI16), and RIG-I are also known to activate caspase-1 [53, 54]. Among the inflammasomes, NLRP3 and AIM2 are the most popular for inducing DNA damage and cytokine release. Interaction of AIM2 with ASC results in the activation of caspase-1 similar to NLRP3 inflammasomes, thus releasing mature cytokines and inducing pyroptosis [55].

A range of stimuli, detected by the innate immune system receptors and sensors have been identified for NLRP3 activation. Under specific conditions, DNA-dependent activators of IRF/Z-DNA binding protein 1 (DAI/ZBP1), DEAD-box polypeptide (DDX), cGAS can mediate NLRP3 inflammasome activation [6, 56]. A recent study showed that ZBP1 promotes pyroptosis and inhibits tumorigenesis [57]. It was reported that DDX3X is important for activating the NLRP3 inflammasome and type I interferon (IFN) responses [58]. Mutations in DDX3X are frequent in subgroups of medulloblastoma, and expression of DDX3X mutants potentiated Wnt pathway signaling [59]. In mouse models of medulloblastoma, Ddx3x knockout increased disease penetrance and reduced tumor latency [60], stimulated by inflammasome activation and cell pyroptosis.

During pyroptotic cell lysis, the release of pro-inflammatory cytokines, IL-1β, and IL-18 trigger a robust immune response within the TME [61, 62]. Apart from these cytokines, dying cells also release a diversity of DAMPs and other mediators, which together act on the innate and adaptive immune components of the immune system, defining the inflammation course of the TME [63, 64]. In human xenograft models of kidney cancer, IL-1β regulates tumor growth and its invasiveness [65], mediated by NLRP3 activation [66].

To this end, Zhivaki et al. have shown that IL-1β was produced by hyperactive dendritic cells in a pyroptosis-independent manner, thereby enabling a durable anti-tumor response. Interestingly, by neutralizing the activity of IL-1β, the antitumor response mediated by hyperactive type 1 DCs (DCs1) can be completely abrogated [67].

In some tumors, the inflammasome effector cytokine activation enhances the production of proangiogenic factors like vascular endothelial growth factor (VEGF) [68]. This is supported by experimental evidence, where the AIM2 cytosolic DNA sensor drives gastric tumor formation which is independent of inflammation [69], by autonomous B-cell mechanisms that operate by suppressing the expression of CXCL16 [70]. In melanoma cancer cells, the improved response following DC vaccination with Aim2 knockout DCs were characterized by increased CD8^+^ T cell recruitment via CXCL10 production [71].

### Pyroptosis in cancer therapies

#### Immunotherapy

Immunotherapeutic approaches, including antibody-mediated therapy (anti-CTLA-4 and anti-PD-1/PD-L1), and chimeric antigen receptor (CAR) T-cell therapy, have improved clinical outcomes following cancer treatment [72, 73]. PD-L1 signals intrinsic to tumor cells regulate the response following DNA damage, suppressing the accumulation of mutations and/or cGAS-STING detection, both of which affect tumor immunogenicity [74]. This promotes anti-PD1 resistance through NLRP3 recruitment of immunosuppressive cell subsets, as observed in the BRAFV600E/PTEN melanoma model [75] with subsequent induction of pyroptosis [76].

Following antigen presentation, the CD8^+^ T cells activate the NLRP3 inflammasomes in DCs, promoting IL-1β maturation and thereby contributing to anti-tumor immunity [77]. In patients undergoing treatment and responding to anti-PD-1 therapy, CD8^+^ T cell frequency along with activated memory CD4^+^ T cells positively correlated with NLRP3 expression [78]. Considering the important role of T cells in tumor immunity, PD-L1 blockade in monocyte-derived DCs resulted in rapid inflammasome activation and CD40L-driven dendritic cell maturation, thus expanding the antigen-specific T cell population [79].

Inflammasome signaling in macrophages drives differentiation of CD4^+^ and Treg (regulatory T-cell) cell populations in pancreatic carcinoma, indicating the possibility of targeting NLRP3 for reprogramming the TME towards an immunogenic phenotype [80]. Tumor-associated NLRP3/IL-1β signaling gives rise to an immunosuppressive environment characterized partly by the regulation of M2-polarized TAMs and a reduction in antitumor CD8^+^ T cells [81]. These results indicate the relationship of the IL-1 signaling pathway to particular genotypes and molecular properties of cancer [82], signifying the importance of IL-1β in modifying the TME.

In melanoma cells, NLRP3/IL-1β activation contributes to the expansion of MDSCs in the TME, thus reducing NK- and CD8^+^ T cell activity and increasing the Treg population [83]. These data support other reports showing that the recruitment of MDSCs may well be due to anti-PD-1-activated T cells, which partially activate the tumor-intrinsic NLRP3 inflammasome [75] in a negative feedback loop. Indeed, the response to immune checkpoint inhibitors may be dependent on the production of chemokines by TAMs [84], which recruit CD8^+^ T and NK- cells following inflammasome activation and pyroptosis [85].

Attacking cancer cells using CD8^+^ cytotoxic T lymphocytes (CTLs) may also result in pyroptosis, facilitating antigen release during the local immune response [28]. However, several features of the TME can influence the propensity of pyroptosis to prime adaptive immune responses [86]. For instance, tumors exhibiting abundant infiltration of macrophages showed that blocking inflammasome activation enhanced suppressive effects on T cells and decreased PD-L1 and indoleamine 2,3-dioxygenase 1(IDO) expression in macrophages which interact with CAR-T and tumor cells [87]. In cytokine release syndrome (CRS), the tumor cell DNA released by pyroptotic cells during CAR-T therapy is internalized by macrophages, activating the inflammasomes and releasing bioactive IL-1β, IL-18, and other pro-inflammatory cytokines [88]. In the process of CAR-T cell therapy, many tumor cells undergo pyroptotic cell death releasing massive amounts of cellular content which enhances the anti-tumor response [89].

Next-generation immune checkpoint inhibitor (ICI) targets such as T cell immunoglobulin and mucin domain-containing (TIM) − 3, is co-expressed with PD-1 on exhausted T cells [90]. Specifically, TIM-3 gene deletion in cDCs promotes reactive oxygen species (ROS) accumulation resulting in downstream inflammasome activation which in turn recruits CD8^+^ TILs, potentiating antigen-specific immunity. However, it has been shown that TIM-3 negatively regulates signaling through IL-1 and IL-18 [91], highlighting the role that these cytokines can have in supporting T-cell responses for antitumor immunity [92].

#### Nanotherapy

Although immunotherapy techniques have shown great promise in targeting tumors, they can enhance the systemic immune response against normal healthy cells leading to unwanted tissue damage. This has led to the development of more targeted therapies such as nanoparticle therapy which allows synthesized nano-range (1-100 nm) particles to specifically target tumor cells and TME, enhance pyroptosis and initiate tumor cell-specific adaptive immune responses [93].

Nanotherapy uses nanoparticles, mainly biomimetic, together with photodynamic therapy and chemotherapy to activate the pyroptotic cell death pathway and boost the anti-tumor response by overcoming toxicity and increasing bioavailability and drug capacity. Cancer cell membrane-based biomimetic nanoparticles (for example, fusing breast cancer membranes onto a polymeric core) target solid tumors both *in-vitro* and *in-vivo*, inducing cancer cell pyroptosis using both photo-treatment and chemotherapy drugs, resulting in the release of pro-inflammatory mediators that activate BMDCs [94].

Several pyroptosis pathways related to mitochondrial modulation can induce PD-L1 secretion [95], through the maturation of DCs and recruiting CD4 and CD8 T + cells, enhancing antitumor efficacy in combination with immunotherapy [96].

To address tumor cell selectivity, Wang and colleagues have utilized a near-infrared (NIR) fluorophore that responds to and induces selective cell pyroptosis in cancer cells overexpressing quinone oxidoreductase isozyme 1 (NQO1). This strategy further boosted antitumor immunity which prolonged survival in a mouse breast cancer model, with the synergizing of the nanocarrier with ɑPD1 [97].

Since mitochondrial stress leads to ROS production which then activates NLRP3 inflammasomes, TME-responsive nanoparticles loaded with chemotherapy drugs followed by laser treatment target mitochondria and activate pyroptosis mediated by GSDME in colon cancer cells [98]. The efficiency of ROS production may be reduced with photodynamic treatment in acidic tumor microenvironments due to decreased tumor penetration depth and reduced Fenton reaction conditions. Persulphate nanoparticles that can produce ROS in any environment along with a surge in intracellular osmolarity and cell lysis caused toxicity in breast and colon cancer cells by activating caspase-1-dependent pyroptosis [99]. Furthermore, these synergistic effects activated anti-tumor immune responses, thereby, reducing tumor metastasis to distant sites, preventing recurrence, and improving survival.

**Fig. 2:**
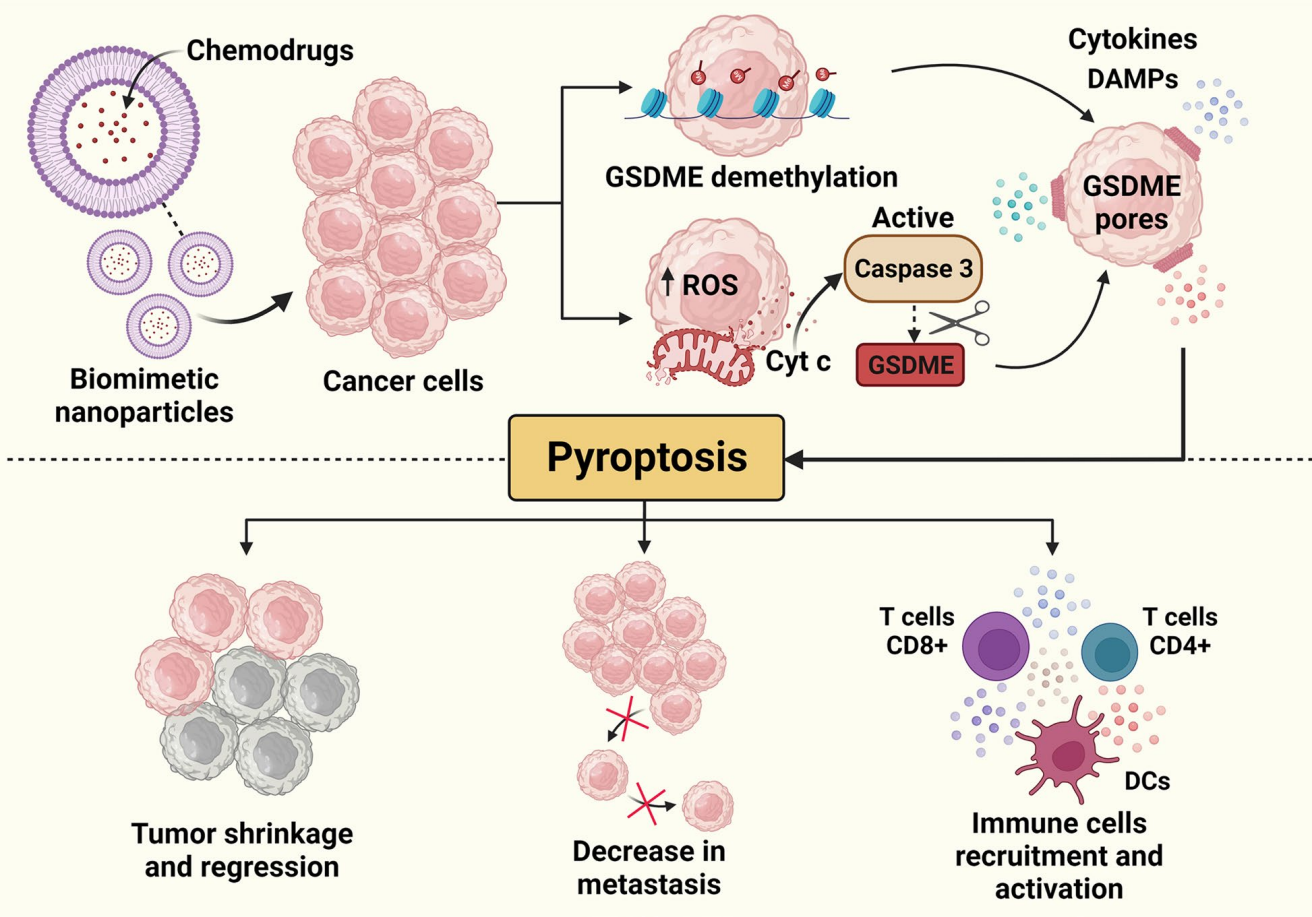
Gasdermin-E mediated pyroptotic cell death and immune cell activation using nanoparticle-based treatments. The association of therapies is essential for a successful anti-cancer treatment. Nanoparticles arise as a key to drug delivery and mitigation of chemotherapy-related collateral effects. The use of nanoparticles to promote chemotherapeutic agents’ delivery associated with the induction of pyroptosis has been explored as an important therapeutic strategy targeting cancer. GSDME is usually silenced in tumors and the encapsulation of some chemo drugs can result in the demethylation of GSDME encoding genes resulting in tumor cell pyroptosis. Another mechanism that has already been described for nanoparticle-based drug delivery is the induction of mitochondrial ROS leading to the liberation of Cytochrome c (Cyt c). These mitochondrial alterations result in the activation of caspase 3 that cleaves GSDME, also culminating in pore formation and pyroptosis. This lytic and inflammatory cell death suppresses tumor growth, decreases metastatic nodules, and enhances immune cells’ activation and anti-tumor immune response

Some chemotherapy drugs can initiate apoptosis in cancer cells by activating caspase-3. However, gasdermin-E, encoded by the DFNA5 (deafness autosomal dominant 5) gene and cleaved by caspase-3 and granzyme B is silenced in some tumors by DNA methylation. This allows tumor cells to escape the local immune response and increase resistance to anticancer therapy. Inhalable nanospheres which incorporate DNA methyltransferases like Decitabine were successfully used to treat lung tumors and reduce metastasis by DFNA-5 gene hypomethylation and gene activation, leading to pyroptosis [100]. Interestingly, combination with chemotherapy drugs can switch cell death from apoptosis to pyroptosis activating CD8 + T cells which kill tumor cells, and dendritic cells which activate immunological memory. Apoptosis to pyroptosis switch was induced in melanoma cells using mitochondrial respiratory inhibitor encapsulated metal-organic nanoparticles which bind specific integrins on melanoma cell membranes and induce tumor cell-specific pyroptosis via elevation of intracellular ROS [101]. These nanoparticles also sensitize tumor cells to immune checkpoint blockade therapy (e.g., ɑPD-1 and ɑPD-L1) (Fig. 2).

Gasdermins induce pyroptosis by forming pores (ca. 10 nm) in the lipid bilayer. Mimicking this process, Chen et al., have produced vesicular DNA nanopores that fuse with the cell membrane in the presence of an acidic TME. Following fusion, these nanopores promote water influx into the cancer cells to induce pyroptosis-like cell lysis which was therapeutically effective in A549 tumor spheroids and hepatoma tumor-bearing mice [102]. This strategy enhanced the anti-tumor effect by increasing tumor-infiltrating T-helper cells, cytotoxic T cells, and NK cells along with enhanced PD-1 expression.

Despite emerging progress in nanoparticle therapy to induce pyroptotic cell death, very limited strategies have succeeded in anti-cancer therapeutic efficiency. Several challenges need to be overcome before the potential clinical transformation of these studies. As an initial prospect, the mechanisms by which these nanoparticles result in pyroptosis-induced tumor cell death using real-time imaging studies can be utilized [93]. Furthermore, the compositions and aspects of the nanoparticles such as stiffness, shape, charge, and immunogenicity can be improved to achieve targeted tumor cell death in both pre-clinical and clinical environments with increased safety, permeability, and systemic bioavailability [103]. Furthermore, the influence of the TME and nanomaterials have to be accounted for when investigating the role of these nano therapies.

### Oncolytic viruses

Oncolytic viruses (OVs) contain the ability to modulate the TME, thus influencing the anti-tumoral immune response [93]. Mechanistically, OVs exert their anti-tumoral function by direct lysis of cancer cells and immune-stimulatory potential. With these potent immunostimulatory properties, OVs can induce cell death including tumor cell pyroptosis, releasing antigens or neoantigens at tumor sites [94].

**Fig. 3:**
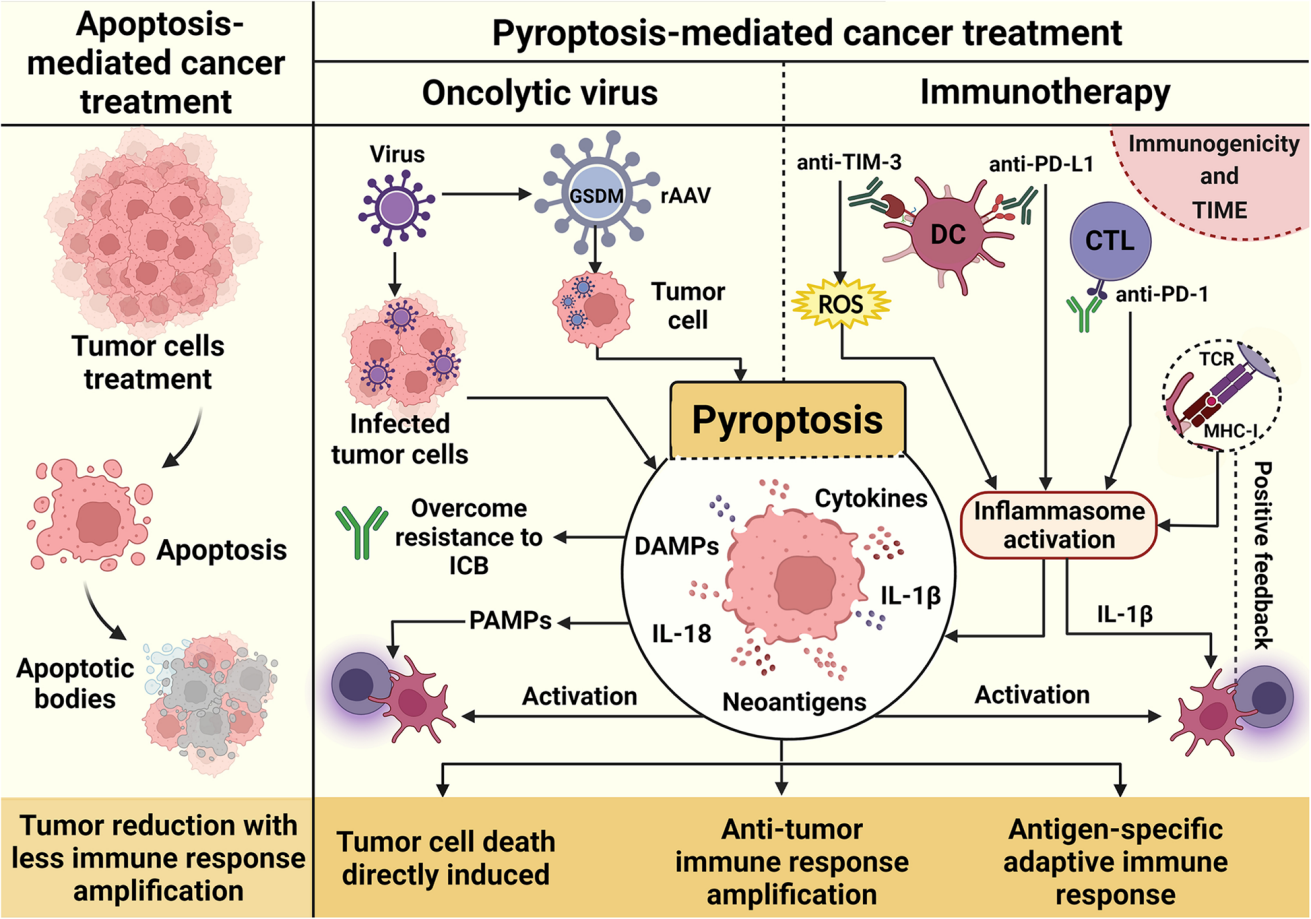
Pyroptosis cell death and therapeutic strategies in cancer. Triggering cell death as an anti-cancer treatment is a central therapeutic goal. Apoptosis-inducing treatments have achieved great results in promoting tumor growth suppression, but with low immunogenic outcomes. Meanwhile, therapeutic approaches that induce pyroptosis have demonstrated interesting results in some models, with immune response amplification and antigen-specific adaptive immune activation. In some contexts, this mechanism enhances the efficacy of therapeutic approaches, such as immunotherapy and the use of oncolytic viruses (OVs). OVs can infect cancer cells and augment their immunogenicity, being an alternative form of drug delivery, directly targeting cancer cells. Recombinant adeno-associated viruses (rAAV) expressing GSDMD are also able to induce successful anti-tumor immunity mediated by this lytic cell death. This therapeutic approach has demonstrated effective results when in combination with other strategies, being an attractive candidate to overcome immune checkpoint blockade (ICB) resistance. Furthermore, the activation of inflammasomes in response to immunotherapy has gained attention, since its activation by anti-TIM-3 and anti-PD-L1 in DCs, as well as anti-PD-1 in cytotoxic T lymphocytes (CTLs), resulting in the cells’ maturation and activation of an effective bridge to adaptive immune responses. Also, the antigen presentation process itself has been involved in NLRP3-dependent maintenance of a positive feedback loop for enhanced immune response. Controversial results have been reported about this topic, as this strategy is extremely sensitive to the immunogenicity and the tumor immune microenvironment (TIME) characteristics, but results point to a promising role of pyroptosis in the development of new anti-cancer therapies

Even more interestingly, in preclinical cancer models, recombinant adeno-associated viruses (rAAVs) expressing GSDM^NT^ infect tumor cells to induce pyroptosis. In addition, the immune checkpoint (PD1/PD-L1) increased the oncolytic effect of rAAV-GSDMD^NT^, stimulating antitumor immunity [96]. In a melanoma model, intradermal injection with active caspase-1 and antigen DNA resulted in pyroptosis, increasing adoptively transferred T cell migration to the tumors [95] (Fig. 3).

#### Dipeptidyl-peptidases inhibitor therapy

Val-boroPro (VbP) non-selectively inhibits post-proline cleavage serine proteases, being active against the dipeptidyl peptidases DPP4,7, 8, and 9 [100]. Inhibition of DPP can activate the immune system to improve the efficacy of immunotherapy in many types of cancer [101]. Inhibition of DPP9 can also activate the NLRP1 inflammasome resulting in caspase-1 activation, cleaved IL-1β, and IL-18 release and pyroptotic cell death in human primary cells and acute myeloid leukemia cell lines [102, 103].

In mouse models of pancreatic cancer, inhibition of DPP9 enhanced NK and T cell infiltration and caused a reduction in tumor growth through inflammasome activation and pyroptosis [85]. Consistent with these findings, DPP4 enhanced the recruitment of CD8^+^ T cells and activated the intrahepatic inflammasome as seen in a mouse model of human hepatocellular carcinoma [104]. Clinical trials report encouraging signs of pharmacologic modulation with Val-boroPro in combination with immunotherapy checkpoints in difficult-to-treat cancers (NCT04171219).

## Conclusion

Recent work has begun to clarify the complex roles of pyroptosis in different aspects of immune function. Regarding the immunomodulatory effects of pyroptosis on tumorigenesis, there are still several unresolved questions. In certain contexts, induction of pyroptosis mediated by DAMPs or by inflammasome agonists can enhance immune cell responses and hinder tumor development, once cytokines, chemokines, cleaved GSDMs, and caspases are released at the same time to respond to tissue damage. On the other hand, pyroptosis through inflammasome-driven secretion of inflammatory mediators could support tumorigenesis [105], through the signaling of cytokines, angiogenic proteins, and growth factors that can lead to tumor proliferation and invasion [106]. Further studies are required to elucidate how mediators of pyroptotic cell death and components of the inflammasomes might affect tumor immunity.

Mediators released during pyroptosis may stimulate M1-polarization of macrophages, promotes maturation of DCs, and activation of CD8^+^ cytotoxic T-lymphocytes (CTLs) in the TME [107], for example, TNF-α and IFN-γ triggered activation of GSDMD, GSDME, caspases 8, 3, 7 and MLKL [108] suggesting the use potential agonists as immunological adjuvants, may increase the efficacy of immunotherapy strategies such as anti-PDL1 antibodies.

Recently, it has been shown that GSDMC may influence the effects of gut-derived microorganisms on anticancer therapy. For example, selective virulence factor deletions in *Listeria monocytogenes* (Lmo) induced GSDMC-dependent pyroptosis modulating intratumoral or systemic T cell responses. Intravenous living bacterial therapy (Lmo@RBC) is also a favored anaerobic colonization strategy that potentiates tumor immune therapy [109].

In general, therapeutic targeting of tumors can be challenging and complex due to tumor heterogeneity and treatment resistance. Modulation of cell death is an avenue that has been explored over the years in cancer therapy where several types of immunogenic cell death (ICD) pathways such as pyroptosis, necroptosis, and ferroptosis (regulated necroptosis) have been researched. Among the ICD forms targeted, pyroptosis has shown more promise in the clinic in comparison to ferroptosis and other ICDs for several reasons. The potential for translation of pyroptosis targeting is mainly due to the involvement of gasdermins which are present only in the pyroptotic cell death pathway [110]. Recently, Wang et al., selectively targeted mouse mammary tumor cells using the Phe-BF_3_ probe, a biorthogonal system that released gasdermins from nanoparticles in tumor cells. Less than 15% of cells undergoing pyroptosis was sufficient to result in T-cell-mediated tumor regression. This evidence further supports the anti-tumor potential of pyroptosis [111]. However, given the complicated role of inflammation in either enhancing or suppressing tumor growth, the components in the pyroptotic pathway along with the timing and the levels of induction need to be strictly controlled during translation into the clinical setting.

Ferroptosis is a distinct type ICD that is iron-dependent and is characterized by an imbalance of the redox state which leads to intracellular ROS production. GPX4 (glutathione peroxidase 4) has been identified by Yang et al., as a key regulator of ferroptosis which inhibited xenograft tumor growth in mice [112]. However, in most human cancers, the p53 tumor suppressor pathway is inactivated which results in an increase in GPX4 and ferroptosis suppression [113].

Evidence also exists that tumor cells enhance oxidative stress as an immune evasion mechanism and therefore, suppress ferroptosis and enhance resistance to this form of cell death [114]. Although ferroptosis has shown some promise in reversing resistance in certain cancer cells, the tumor cell populations that it targets are still unclear and more research is needed as to what factors are released during ferroptosis, cancer cell sensitivity to ferroptosis inducers, mechanisms involved in this form of cell death and in vivo biomarkers of ferroptosis before it’s practical application in the clinic. Furthermore, ferroptosis is involved in pathological cell death in various degenerative and ischemic diseases, which necessitates treatment that specifically targets ferroptosis in cancer cells without causing systemic toxicity [115–117].

On the other hand, one of the challenges of the pyroptosis-based therapeutic approach to cancer is that, in different tumors, the expression and function of its activators can be altered by a variety of stimuli, and mutational and epigenetic mechanisms. Considering the number of therapeutic targets and strategies, as well as the different immunological landscapes, among patients, pre-clinical studies in organoid models [118] and humans are essential to explore the power of gasdermins, cytokines, and their receptors released during pyroptosis.

In addition, the different therapeutic strategies against cancer, including immunotherapy, oncolytic virus therapy, and nanoparticle-based therapies could be guided to trigger and regulate pyroptotic cell death in cancer cells, leading to tumor-shrinking or remission. These pyroptosis-based cancer therapies may open up fresh avenues for targeted cancer therapy approaches in the future and their translation into the clinic.

## Data Availability

Not applicable.

## Abbreviations

AIM2: Absent in melanoma 2
ATP: Adenosine-triphosphate
cGAS: Cyclic guanosine monophosphate-adenosine monophosphate synthase
CLRs: Type lectin receptors
CRS: Cytokine release syndrome
CTLA-4: Cytotoxic T-lymphocyte antigen 4
CTLs: Cytotoxic T lymphocytes
DAMPs: Damage-associated molecular patterns
DCs: Dendritic cells
DDX: DEAD-box polypeptide
DFNA5: Deafness autosomal dominant 5
DPP: Dipeptidyl peptidases
GSDMA: Gasdermin A
GDMB: Gasdermin B
GSDMC: Gasdermin C
GSDMD: Gasdermin D
GPX4: Glutathione peroxidase 4
ESCRT: Endosomal sorting complex required for transport
ICD: Immunogenic cell death
ICI: Immune checkpoint inhibitor
IDO: Indoleamine 2,3-dioxygenase 1
IFNγ: Interferon- γ
IL-1β: Interleucin 1β
IL-18: Interleucin 1β
IRF2: Interferon regulatory transcription factor-2
LMO: *Listeria monocytogenes*
LPS: Lipopolysaccharide
MDSCs: Myeloid-derived suppressor cells
NIR: Near-infrared
NKs: Natural killer
NLRs: Leucine-rich repeat–containing receptors
NLRP3: NOD-, LRR- and pyrin domain-containing protein 3
NODs: Nucleotide-binding oligomerization domain
NQO1: Quinone oxidoreductase isozyme 1
OVs: Oncolytic viruses
PAMPs: Pathogen-associated molecular patterns
PD-1: Programmed death receptor 1
PRRs: Pattern recognition receptors
rAAVs: Recombinant adeno-associated viruses
RIG-I: Retinoic acid-inducible gene I
ROS: Reactive oxygen species
TAMs: Tumor associated macrophages
TIM-3: T cell immunoglobulin and mucin domain-containing
TLRs: Toll-like receptors
TME: Tumor microenvironment
VbP: Val-boroPro
VEGF: Endothelial growth factor
